# Peripapillary Choroidal Neovascular Membrane: A Case Report

**DOI:** 10.7759/cureus.57050

**Published:** 2024-03-27

**Authors:** Rana S Alojair, Muhammed Amer, Abdulmajeed Alkhathami, Ibrahim N Asiri

**Affiliations:** 1 Department of Ophthalmology, Armed Forces Hospital Southern Region, Khamis Mushait, SAU; 2 Department of Ophthalmology, University of Bisha, Bisha, SAU

**Keywords:** saudi arabia, intravitreal injection, case report, retina, peripapillary choroidal neovascular membrane

## Abstract

Peripapillary choroidal neovascular membrane (PCNM) is an abnormal growth of blood vessels beneath the retina near the optic disc. We report a case of a 60-year-old Saudi female with a history of hypertension, hypothyroidism, and epilepsy who presented to the emergency room (ER), reporting a sudden decrease in vision over the past month. Ophthalmic examination revealed reduced visual acuity. The patient received aflibercept via intravitreal injection every four weeks. On follow-up, she reported improvement in symptoms. It has been shown that intravitreal anti-vascular endothelial growth factor (VEGF) not only preserves visual acuity but also produces anatomic improvement when used alone or in conjunction with other therapeutic modalities like photodynamic therapy, laser photocoagulation, and subretinal surgery, as PCNM is aberrant blood vessel growth under the retina. Only a few cases have been recorded in Saudi Arabia; we report this case to emphasize the importance of diagnosis and timely treatment with anti-VEGF.

## Introduction

The peripapillary choroidal neovascular membrane (PCNM) is a type of choroidal neovascular membrane (CNVM), a collection of aberrant blood vessels that emerge from the choroid through a rupture in the Bruch's membrane and are located near the optic disc. PCNM can result in severe visual loss [1]. Patients with exudative age-related macular degeneration (AMD) who are older than 50 years old are usually the ones who experience PCNM [2]. These abnormal blood vessels can cause vision loss or distortion by leaking fluid and blood into the surrounding tissue. Significant vision loss can result from the spread of blood or fluid into the macula. Common symptoms include blurred or distorted central vision, abnormal blood vessels that can disrupt the normal structure of the retina, leading to blurry or distorted vision, especially in the central part of the visual field, and metamorphopsia. It is a common symptom in CNVM due to the distortion of the retinal tissue. A central scotoma is an area of reduced or absent vision in the central part of the visual field due to abnormal blood vessels affecting the macula, which is responsible for detailed central vision [3]. Diagnosing peripapillary CNVM typically involves a comprehensive eye examination that includes a dilated fundus examination, optical coherence tomography (OCT), and fluorescein angiography.

To our knowledge, few cases have been reported in Saudi Arabia; this is the first case to be reported from the southern region. We report this case to disseminate valuable clinical information, enhance medical knowledge, and potentially guide future research and treatment strategies for peripapillary choroidal neovascular membranes.

## Case presentation

A 60-year-old Saudi woman with a history of hypertension, hypothyroidism, and epilepsy presented with a sudden decrease in vision for the past month with no other associated symptoms. She denied experiencing any past trauma, photophobia, ocular discharge, tears, or eye surgery. There was no history of pain. Negative systemic history and no relevant family history were reported. Ophthalmic examination revealed reduced visual acuity and a relative scotoma in the affected eye. The fundus examination and optical coherence tomography (OCT) indicated a peripapillary CNVM associated with subretinal fluid and hyperreflective material. Fluorescein angiography confirmed the presence of active leakage from the neovascular membrane. Ophthalmological examination is shown in Table 1. 

**Table 1:** Ophthalmology examination in both eyes cc - with correction

Visual acuity (VA)	Oculus dexter (OD)	Oculus sinister (OS)
	6/60 cc	6/24 cc
Refraction	sphere +4.00, cylinder -3.50, axis 10 (6/18)	sphere +5.50, cylinder -5.00, axis 170 (6/9)
Intraocular pressure (IOP)	17 mmHg	16 mmHg
Pupil	Equally round and reactive to light (RRR)	RRR
Slit lamp examination	Anterior segment: within normal limits, lens: clear	Anterior segment: within normal limits, lens: clear

A funduscopic examination of the right eye demonstrated juxtapapillary/peripapillary subretinal neovascularization at the inferotemporal arcade level. This was linked to a hard exudate involving the optic disc. The left eye showed a healthy disc and a flat retina. 

**Figure 1:**
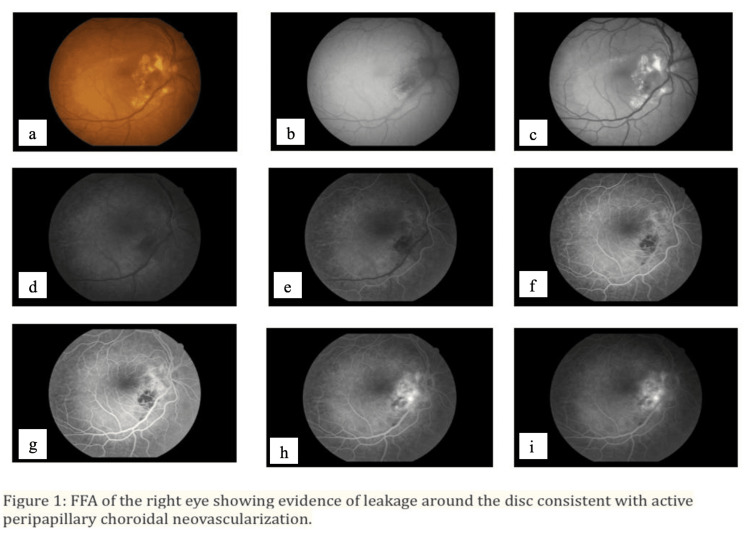
The right eye's fundus fluorescein angiography (FFA) reveals leaking around the disc, which is consistent with active peripapillary choroidal neovascularization. (a) The right eye's color fundus photo upon presentation shows signs of leaking surrounding the disc, which is consistent with active peripapillary choroidal neovascularization, (b) late phase: dye diffusion outside the lesion's boundaries, (c)–(e) On the right eye's early and late fluorescein angiography, a choroidal neovascular membrane is visible next to the disc, (f)-(i) exhibits a hypoflourescence patch where bleeding has obstructed the backdrop.

**Figure 2:**
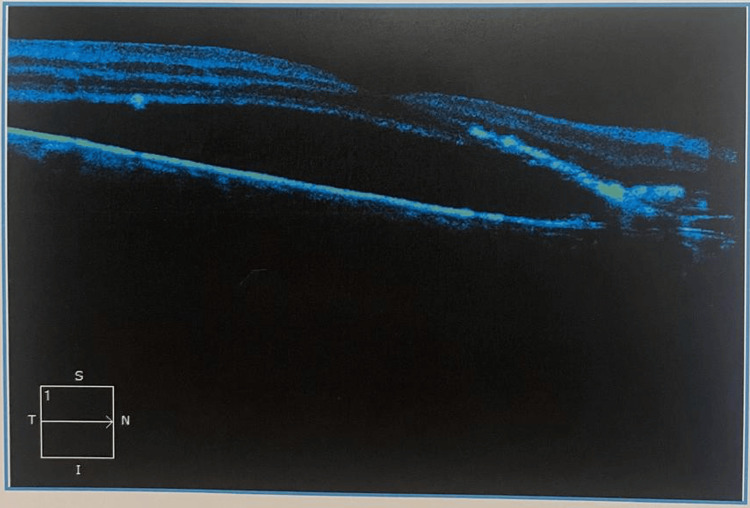
Optical coherence tomography (OCT) of the right eye showing marked subretinal fluid at the retinal pigment epithelium (RPE)

In order to rule out antecedent causes, a screening blood test was performed. The findings of the Mantoux test, rheumatoid factor, toxoplasma, syphilis, rubella, cytomegalovirus, and herpes simplex (TORCH) titers, rapid plasma reagin (VDRL), HIV, and serum calcium were all normal. The patient underwent fundus fluorescein angiography (FFA), as shown in Figure 1, and OCT on both eyes, as shown in Figure 2.

The plan entailed undergoing intravitreal injections (IVI) three times over a period of three months. After obtaining written informed consent from the patient, she was treated with intravitreal 2 mg (0.05 mL) aflibercept every four weeks for three doses. On the follow-up, she reported improvement in symptoms after receiving three doses. Ophthalmology examination after the third dose is shown in Table 2.

**Table 2:** Ophthalmological examination after receiving the treatment

Visual acuity (VA)	OD	OS
	6/9-	6/15
Slit lamp examination	Early cataract changes	Early cataract changes
Fundus examination	Exudates around the optic disc, flat retina	normal optic disc, flat retina

**Figure 3:**
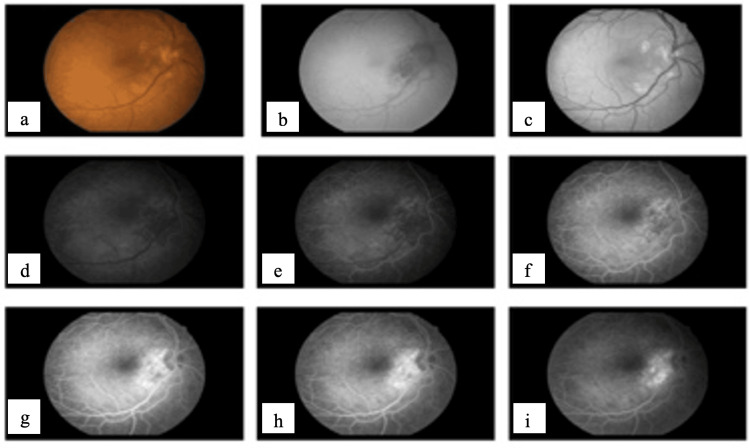
Fundus fluorescein angiography (FFA) of the right eye showing evidence of window defect around the disc consistent with inactive peripapillary choroidal neovascular membrane (CNVM) (a) Color fundus photograph of the right eye after receiving three doses of (IVI) showing resolution of the subretinal fluid and hemorrhage; (b)-(i) following the third aflibercept injection showing neovascular membrane regression and subretinal fluid absence.

**Figure 4:**
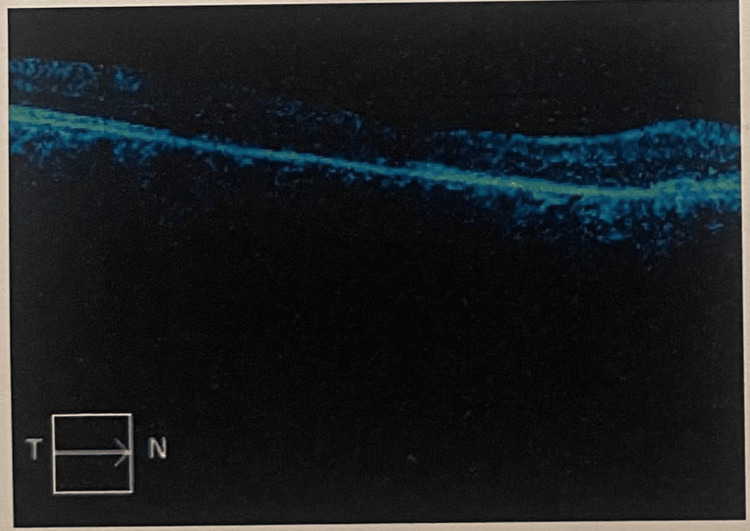
Optical coherence tomography (OCT) of the right eye showing resolution of the subretinal fluid at three months follow-up

An FFA was done, which showed the inactivity of previously active CNVM (Figure 3) with normal OCT (Figure 4). The patient is now on regular follow-up.

## Discussion

Symptoms of peripapillary CNVM can include scotomas, metamorphopsia, and reduced visual acuity. Diagnosing these vague symptoms can be challenging, as they can coexist with other macular and optic nerve disorders. Differential diagnoses include age-related macular degeneration, myopic CNVM, optic disc drusen, and optic neuropathies. Peripapillary CNVM can be diagnosed and assessed using a variety of imaging techniques. A fundus examination provides significant information about the position, dimensions, and texture of the neovascular membrane. OCT makes it possible to image choroidal and retinal abnormalities in great detail, including subretinal fluid, intraretinal cysts, and hyperreflective material. Our clinical diagnosis was verified by OCT and fluorescein angiography.

The peripapillary subretinal neovascular membrane can be distinguished from other illnesses with comparable clinical presentations with the aid of indocyanine green (ICG) angiography, especially polypoidal choroidal vasculopathy. Moreover, it may provide CNVM images with improved occult CNVM delineation using blood or serous fluid. However, since the advent of anti-VEGF medication, the significance of ICG in assessing the clinical features of CNVM has significantly decreased [4]. The leaking pattern and the site of neovascularization can be determined with the aid of fluorescein and indocyanine green angiography. Intravitreal anti-vascular endothelial growth factor (anti-VEGF) injections, photodynamic therapy, and laser photocoagulation are possible treatment options. Early intervention is essential to enhance long-term outcomes and prevent irreparable sight loss. The prognosis for the idiopathic neovascular membrane is often better than that of AMD since it is usually unilateral, and occasionally spontaneous remission may happen [5]. Choroidal neovascularization is a major contributor to vision loss and a symptom of numerous disorders affecting the retinal pigment epithelium, choroid, and Bruch membrane [3]. Clinically, choroidal neovascular membranes next to discs that produce subretinal bleeding, fluid, and/or exudate indicate peripapillary CNV [1]. Subretinal fluid, intraretinal fluid, subretinal hemorrhage, and subretinal hyperreflective lesion are OCT characteristics that may be indicative of CNV. On fluorescein angiography, a well-defined CNV appears as a lacy network of capillary plexuses that leaks as the angiogram goes on [6]. Anti-VEGF agents (aflibercept) have been used as a treatment option for peripapillary CNV associated with optic nerve head drusen. These work by reducing the levels of VEGF, thereby inhibiting the growth of abnormal blood vessels and reducing fluid leakage in the retina. Treatment with anti-VEGF agents typically involves regular injections into the eye. The frequency of injections and the duration of treatment may vary depending on the individual case and the response to the medication [7]. Similar to previous treatment modalities, PCNV responds to intravitreal bevacizumab with a reduction in retinal fluid and improvement or preservation of vision [3]. When used in conjunction with other therapy modalities such as photodynamic therapy, laser photocoagulation, and subretinal surgery, intravitreal bevacizumab has been demonstrated to produce anatomic improvement in addition to maintaining visual acuity or even improvement comparable to that of other treatment modalities [8].

## Conclusions

In the absence of risk factors, the peripapillary subretinal neovascular membrane is a disorder that seldom affects old, healthy individuals. This case report emphasizes how crucial it is to conduct a thorough ocular examination using a range of imaging modalities to provide an accurate diagnosis. Frequent follow-ups to monitor therapy responses and avoid visual damage are essential. Even though our case report showed that anti-VEGF is safe and effective for managing this case after three doses, long-term comparison, and follow-up studies are required to ascertain the anti-VEGF's safety, effectiveness, and potential side effects.
